# Glaucomatous Visual Field Defect Severity and the Prevalence of Motor Vehicle Collisions in Japanese: A Hospital/Clinic-Based Cross-Sectional Study

**DOI:** 10.1155/2015/497067

**Published:** 2015-04-01

**Authors:** Takeshi Ono, Kenya Yuki, Ryo Asaoka, Keisuke Kouyama, Takayuki Abe, Sachiko Tanabe, Kazumi Fukagawa, Miki Uchino, Masaru Shimoyama, Yoko Ozawa, Naoki Ozeki, Daisuke Shiba, Kazuo Tsubota

**Affiliations:** ^1^Department of Ophthalmology, Keio University School of Medicine, Tokyo 160-8582, Japan; ^2^Department of Ophthalmology, Graduate School of Medicine, The University of Tokyo, Tokyo 113-8655, Japan; ^3^Clinical Research Centre, Keio University School of Medicine, Tokyo 160-8582, Japan; ^4^Tanabe Eye Clinic, Yamanashi 400-0177, Japan; ^5^Iidabashi Eye Clinic, Tokyo 102-0072, Japan

## Abstract

*Purpose*. This study examined the association between the severity of visual field defects and the prevalence of motor vehicle collisions (MVCs) in subjects with primary open-angle glaucoma (POAG). *Methods*. This is a cross-sectional study. Japanese patients who have had driver's licence between 40 and 85 years of age were screened for eligibility. Participants answered a questionnaire about MVCs experienced during the previous 5 years. Subjects with POAG were classified as having mild, moderate, or severe visual field defect. We evaluated associations between the severity of POAG and the prevalence of MVCs by logistic regression models. *Results*. The prevalence of MVCs was significantly associated with the severity of POAG categorized by worse eye MD (control: 30/187 = 16.0%; mild POAG: 17/92 = 18.5%; moderate POAG: 14/60 = 23.3%; severe POAG: 14/47 = 29.8%; *P* = 0.025, Cochran-Armitage trend test). Compared to the control group, the adjusted OR for MVC prevalence in subjects with mild, moderate, or severe POAG in the worse eye was 1.07 (95% CI: 0.55 to 2.10), 1.44 (95% CI: 0.68 to 3.08), and 2.28 (95% CI: 1.07 to 4.88). *Conclusions*. There is a significant association between the severity of glaucoma in the worse eye MD and the prevalence of MVCs.

## 1. Introduction

Motor vehicle collisions (MVCs) are among the most common serious public health concerns in the world: it is estimated that each year 1 to 2 million people die in MVCs and another 50 million are injured, costing the global community about US$518 billion [1].

Glaucoma is the second leading cause of blindness in the world, affecting approximately 5 million adults globally and damaging both peripheral and central vision [2]. Age is a significant risk factor for primary open-angle glaucoma (POAG) [3]. As the elderly population and, concomitantly, the number of elderly drivers continue to grow in both developed and developing countries, ever more elderly drivers with glaucomatous visual field defects are on the road. Glaucoma patients have been reported to have problems in everyday vision related activities such as search [4] and face recognition [5]. Smith et al. reported that, in comparison to control patients, glaucoma patients have difficulty finding objectives in photographs of everyday scenes [4]. Since driving is really a vision-dependent task, individuals with glaucoma may have a higher risk of being involved in MVCs.

Several studies have investigated associations between glaucoma and MVCs [6–13]. Haymes et al. compared 47 normal, healthy control subjects and 48 with glaucoma and concluded that individuals with glaucoma were more than 6 times more likely to be involved in MVCs [13]. However, the associations between the severity of glaucomatous visual field defects and the prevalence of MVCs have not been clarified, so we examined these associations in subjects with POAG.

## 2. Subjects and Methods

This study's procedures conformed to the tenets of the Declaration of Helsinki and to national (Japanese) and institutional (Keio University School of Medicine) regulations. The study was approved by the Ethics Committee of the Keio University School of Medicine (number 2010293). All study subjects gave informed, written consent prior to being enrolled.

### 2.1. Study Design and Subject Enrolment

Japanese patients between 40 and 85 years of age who visited Keio University Hospital (Tokyo, Japan), Iidabashi Eye Clinic (Tokyo, Japan), or Tanabe Eye Clinic (Yamanashi, Japan) between May 1, 2011, and November 30, 2011, were screened for eligibility for this cross-sectional study. Patients with POAG and control subjects were screened at these institutions' glaucoma clinics and general outpatient clinics, respectively.

### 2.2. Evaluation of Subjects with Glaucoma

Patients with glaucoma were screened for eligibility using a battery of ophthalmic examinations, including slit-lamp biomicroscopy, funduscopy, gonioscopy, intraocular pressure measurements by Goldmann applanation tonometry, and visual field examination with a Humphrey visual field analyser (HFA) and the 24-2 Swedish Interactive Threshold Algorithm Standard Strategy (Carl Zeiss Meditec, Dublin, CA). The findings were analyzed by S. T. and K. Y. whom subspecialize in glaucoma. The reliability of the findings was confirmed to be high, with less than a 20% fixation loss rate and less than a 15% false-positive rate [14].

POAG was diagnosed when three findings were present: (1) glaucomatous optic cupping, represented by notch formation, generalized cup enlargement, a senile sclerotic or myopic disc, or nerve-fibre layer defects; (2) glaucomatous visual field defects, defined according to Anderson and Patella's criteria (a cluster of 3 or more points in the pattern deviation plot within a single hemifield (superior or inferior) with a *P* value < 5%, one of which must have a *P* value < 1%; [15]); and (3) an open angle observed on gonioscopy.

### 2.3. Evaluation of Control Subjects

Most of these patients were seen for an annual eye check-up or for outer adnexal disease. Control subjects were evaluated by ophthalmic examination, including best corrected visual acuity (BCVA) measurements, autorefractometry, slit-lamp biomicroscopy, funduscopy, and intraocular pressure measurements using Goldmann applanation tonometry or a noncontact tonometer. The findings were analysed by S. Tanabe and K. Yuki. Control subjects had to be free of ocular fundus disease that might affect visual function and to have a decimal BCVA in both eyes of 0.7 or more.

### 2.4. Exclusion Criteria

Subjects were excluded if they had an ophthalmologic disease other than POAG that could potentially compromise visual acuity or contribute to visual field loss, such as secondary glaucoma or age-related macular degeneration. Subjects were also excluded if they had a decimal BCVA of less than 0.7, if they did not have a driver's license or drove 1 kilometre or less per week, or if they had a mental disorder that prevented them from understanding the questionnaire. Of the 943 consecutive subjects screened, 557 were excluded. The reasons for excluding subjects were as follows (the numbers in parentheses indicate the number of subjects excluded): being younger than 40 (53), being older than 85 (56), refusal to participate (10), dementia (2), low visual acuity (26), secondary glaucoma (61), primary angle-closure glaucoma (15), postretinal-detachment (20), diabetic retinopathy (36), bullous keratopathy (2), age-related macular degeneration (5), other ocular diseases (7), never having a driver's license (175), driving less than 1 kilometer per week (89).

### 2.5. Evaluation of Motor Vehicle Collisions

All study participants answered the following questionnaire in Japanese (translated):

1. Do you have a driver's license? (Yes/No/Previously)
2. How long have you driven/did you drive a car? (_ _ _years)
3. How many kilometres per week do you normally drive? (_ _ _km)
4. Have you been involved in one or more traffic accidents in the past five years, including a single-car or minor accident, in which you were driving the car? (Yes/No)
5. How many traffic accidents have you ever been involved in, in the past five years? (_ _ _)

Demographic information recorded for all subjects included age, sex, height, weight, alcohol intake (yes/no), smoking history (yes/no/previous), current and previous illnesses (e.g., systemic hypertension, diabetes mellitus, depression, and brain infarction), and medical history, including oral medications such as sleeping aids, antihypertensive drugs, or tranquilizers.

### 2.6. Integrated Binocular Visual Field

A binocular integrated visual field (IVF) was calculated for each patient by merging a patient's monocular HFA VFs, using the “best sensitivity” method, where the IVF total deviation (TD) at each point was calculated using the maximum TD (least negative) value from each of the two overlapping points, as if the subject was viewing the field binocularly [16]. The IVF MD was calculated as the mean of 52 TD values across the visual field. We were unable to obtain IVF data for 8 POAG subjects.

### 2.7. Grading Glaucoma Severity

For this study, we defined mild POAG as a visual field defect corresponding to a mean deviation (MD) of −6 dB or better, moderate POAG as an MD between −6 and −12 dB, and severe POAG as an MD of −12 dB or worse [17]. For each patient, we determined POAG severity for the worse eye (the more negative MD), the better eye (the more positive MD), and the IVF.

### 2.8. Adjusting for Age

In our previous report, the results could have been biased by significant age differences between the control group and the three POAG groups [12]. In this study, the average ages of the groups were compared by ANOVA at the end of each month. When significant age differences were found between the groups, we matched the ages by changing the screening criteria in the youngest group from 40–85 years of age to 45–85 years of age. This adjustment was necessary only in the mild glaucoma group and was made only in the months of September and November.

### 2.9. Statistical Analysis

Descriptive statistics were calculated for the demographic, medical, and visual-function variables. The homogeneity of distribution between the control and POAG groups was examined by ANOVA, Kruskal-Wallis test, chi-square test, or Fisher's exact test, depending on the variables. The association between POAG severity and the prevalence of MVCs was evaluated with the Cochran-Armitage trend test.

Adjusted ORs and 95% CIs for the prevalence of MVCs were estimated with logistic regression models to examine the effects of the following confounding factors on unadjusted results, by the forced-entry method: glaucoma severity (control, mild, moderate, and severe POAG groups), age, sex, the presence of diabetes mellitus, the proportion of alcohol drinkers in the group, the BCVA in the better eye, and the distance driven each week.

The accident rate, which represents the number of MVCs per 10,000 km driven, was calculated by the number of MVCs (question 5) divided by the (average distance driven per week (question 3) × 52 (weeks/year) × 5 years) × 10,000.

Associations between the number of MVCs and POAG severity, and between the accident rate and POAG severity, were evaluated by Jonckheere-Terpstra tests.

A *P* value less than 0.05 was considered statistically significant. Decimal visual acuity was converted to LogMAR visual acuity for analysis. All data were analysed with IBM SPSS statistics software version 21.0 (IBM Japan, Tokyo, Japan).

## 3. Results

We enrolled 199 consecutive POAG patients and 187 consecutive control subjects in this study. The POAG patients were divided into three groups according to the severity of POAG in the worse eye, better eye, and IVF. All participants were of Asian ethnicity. The subjects' demographic characteristics are shown grouped by worse eye MD in Table 1, by better eye MD in Table 2, and by IVF MD in Table 3. No significant differences were observed in age, sex, prevalence of diabetes mellitus, or the number of comorbid illnesses among controls and POAG groups when categorized by the MD in the worse eye, better eye, or IVF. When grouped by MD in the worse eye, there were significant differences in BCVA in the worse eye between the control and POAG groups.

The prevalence of MVCs did not differ significantly between the control group and the three POAG groups combined (control subjects: 30/187 (16.0%); POAG subjects: 45/199 (22.6%); *P* = 0.12). However, there was a statistically significant association between the prevalence of MVCs and POAG severity in the worse eye (*P* = 0.025, Cochran-Armitage trend test, Table 4). We did not observe a significant association between the prevalence of MVCs and POAG severity in the better eye (*P* = 0.12) or IVF (*P* = 0.27; Cochran-Armitage trend test, Table 4).

Adjusted ORs and 95% CIs were estimated with logistic regression models. Compared to the control group, the adjusted ORs for the prevalence of MVCs in subjects with mild, moderate, or severe POAG in the worse eye were 1.07 (95% CI: 0.55–2.10), 1.44 (95% CI: 0.68–3.08), and 2.28 (95% CI: 1.07–4.88), respectively, as shown in Table 5. However, no significant association was observed in subjects with mild, moderate, or severe POAG categorized by POAG severity in the better eye or IVF (Table 5).

The mean number of MVCs per group in the past five years was 0.19 ± 0.48 (interquartile range: 0) in the control group, 0.30 ± 0.77 (interquartile range: 0) in the mild glaucoma group, 0.28 ± 0.56 (interquartile range: 0) in moderate glaucoma group, and 0.36 ± 0.61 (interquartile range: 1) in the severe glaucoma group, when subjects were grouped by POAG severity in the worse eye. This trend is statistically significant (*P* = 0.03, Jonckheere-Terpstra test). When subjects were grouped by POAG severity according to the MD in the better eye or the IVF, the number of MVCs was 0.19 ± 0.48 (interquartile range: 0) in the control group, 0.31 ± 0.69 (interquartile range: 0) and 0.35 ± 0.70 (interquartile range: 0) in the mild glaucoma group, 0.29 ± 0.55 (interquartile range: 0.5) and 0.15 ± 0.37 (interquartile range: 0) in the moderate glaucoma group, and 0.33 ± 0.65 (interquartile range: 0.5) and 0.33 ± 0.82 (interquartile range: 0) in the severe glaucoma group (*P* = 0.08, *P* = 0.11, resp., Jonckheere-Terpstra test).

**Figure 1:**
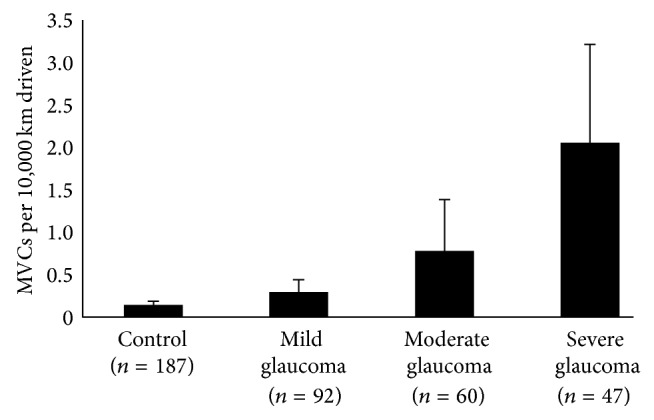
The association between the number of MVCs per 10,000 km driven and glaucoma severity, grouped by worse eye. The number of MVCs went up when the severity of the glaucoma, grouped by worse eye, increased. The trend was statistically significant (*P* = 0.03, Jonckheere-Terpstra test). MVC (motor vehicle collision). Error bar: standard error.

**Figure 2:**
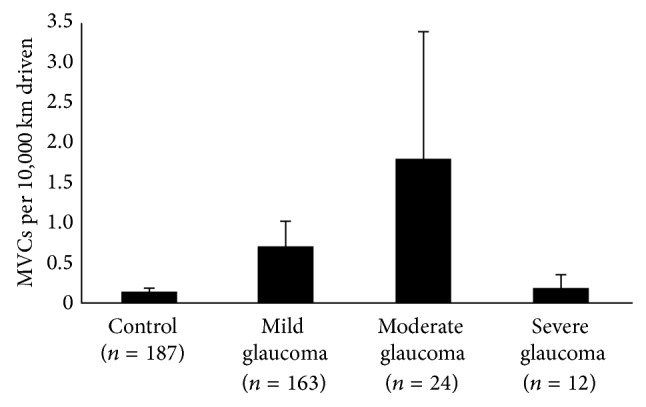
The association between the number of MVCs per 10,000 km driven and glaucoma severity, grouped by better eye. No significant association was observed between the accident rate and glaucoma severity, grouped by better eye (*P* = 0.08, Jonckheere-Terpstra test). MVC (motor vehicle collision). Error bar: standard error.

**Figure 3:**
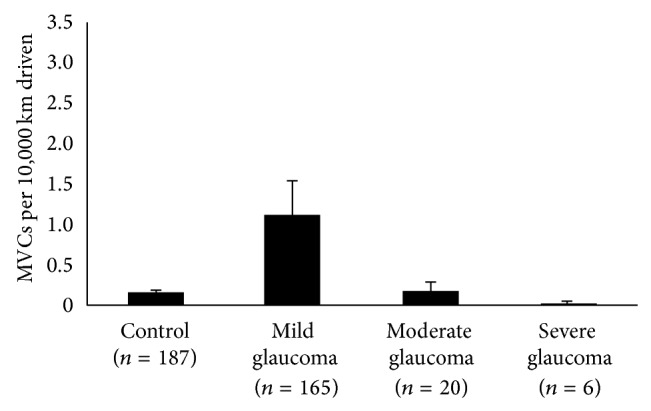
The association between the number of MVCs per 10,000 km driven and glaucoma severity, grouped by IVF. No significant association was observed between the accident rate and glaucoma severity, grouped by IVF (*P* = 0.12, Jonckheere-Terpstra test). MVC (motor vehicle collision); IVF (integrated visual field). Error bar: standard error.

The accident rates are shown in Figures 1 to 3. When subjects were grouped by POAG severity in the worse eye, the accident rate (the number of MVCs per 10,000 km driven) was 0.1 ± 0.5 in the control group, 0.3 ± 0.1 in the mild glaucoma group, 0.8 ± 0.5 in the moderate glaucoma group, and 2.1 ± 8.0 in the severe glaucoma group (mean ± standard deviation). The accident rate in the severe glaucoma group was significantly higher than that in the control group (*P* < 0.001 ANOVA, Tukey post hoc test). However, no significant difference was observed among the four groups (ANOVA; *P* = 0.06 and *P* = 0.05, resp.) when grouped by POAG severity in the better eye or IVF. The trend was statistically significant (*P* = 0.03, Figure 1, Jonckheere-Terpstra test) when POAG severity was categorized by MD for the worse eye but not by MD for the better eye (*P* = 0.08, Figure 2) or IVF (*P* = 0.12, Figure 3).

After multivariable adjustment, younger age (ORs 0.74 (95% CI: 0.60–0.90) per 10-year increment, *P* = 0.01) and greater distance driven per week (ORs 1.02 (95% CI: 1.01–1.04) per 10 km, *P* = 0.008) were associated with MVCs. However, the BCVA in the better eye (ORs 0.94 (95% CI: 0.87–1.01) LogMAR per 0.1 increment, *P* = 0.16), the presence of diabetes mellitus (ORs 1.56 (95% CI: 0.76–3.17), *P* = 0.20), and the proportion of subjects in the group who drank alcohol (ORs 0.95 (95% CI: 0.53–1.69), *P* = 0.84) were not associated with MVCs after multivariable adjustment for glaucoma severity in the worse eye. Younger age and a greater distance driven per week were also associated with MVCs after multivariable adjustment for glaucoma severity categorized by the MD of the better eye or IVF.

## 4. Discussion

In this study, we showed that the severity of glaucomatous visual field defects is significantly associated with the prevalence of MVCs. The adjusted OR for MVCs was 2.5 times higher for subjects with severe POAG in the worse eye than for control subjects.

Our results are compatible with findings from our former study [12]. Our previous report compared the prevalence of self-reported MVCs among normal, healthy control subjects (*n* = 121) and those with mild (*n* = 50), moderate (*n* = 51), or severe POAG (*n* = 20). In that study, we found that subjects with a severe visual field defect in the worse eye were more likely to be involved in MVCs (OR 9.9 (95% CI, 2.1–47.8), control as reference). In a well-designed, nested case-control study in patients with glaucoma, McGwin Jr. et al. compared the severity of visual field defects between subjects who had or had not been involved in MVCs, using Advanced Glaucoma Intervention Study (AGIS) scores, and concluded that patients with moderate or severe visual field defects (AGIS score 12–20) in the worse eye had a significantly increased risk for MVCs (OR 3.6 (95% CI 1.4–9.4) and OR 4.4 (95% CI 1.6–12.4), resp.) [8]. These studies are compatible with our findings that the MD in the worse eye is associated with MVCs.

Our findings are also compatible with findings from another study on the association between driving performance and visual field defects: Haymes et al. reported that the MD in the worse eye is correlated with real-world driving performance [18]. Multivariable analysis showed that patients with glaucoma and an MD < −4 dB in the worse eye were more than 4 times more likely to have the driving instructor intervene during real-world driving compared to those with better visual fields and that a poor MD was the predominant cause of failure to see and yield to a pedestrian [18]. It is reasonable to speculate that poor driving performance may lead to MVCs. These results support our finding that a more severe visual field defect in the worse eye is associated with more MVCs.

The association between the accident rate, defined as the number of MVCs per 10,000 km driven, and glaucoma severity is especially interesting. It has been suggested that a worse glaucomatous visual field is associated with driving cessation and limitations [19]. Therefore, the accident rate is one of the most accurate indicators for the possibility of being involved in MVCs. We found that the accident rate was 14 times higher for subjects with a severe visual field defect in the worse eye than for control subjects; this difference is statistically significant. This result suggests that it is important for ophthalmologists to notify patients with severe glaucoma that if they drive, they may have a higher risk for MVCs.

We found that younger age was associated with MVCs, even after adjusting for distance driven. It has been reported that the relationship between age and MVCs forms a U-shape curve [20, 21], and middle-aged subjects (40–50 years old) are the safest drivers. The reason for this discrepancy between our study and previous studies is unclear. However, we excluded subjects with low visual acuity, age-related macular degeneration, and other ocular diseases associated with older age, and these visual impairments are risk factors for MVCs [11, 22, 23]. The higher risk found for MVCs in younger subjects in this study may reflect the exclusion of older subjects with age-related visual impairments other than glaucoma.

We also found that greater driving distances were associated with MVCs. Theoretically, subjects who drive greater distances are exposed to a higher risk of being involved in MVCs than those who drive fewer kilometres. Our results suggest that, among drivers with glaucoma, youngest drivers with a severe visual field defect in the worse eye who drive long distances may have the highest risk of MVCs.

In this study, we did not find significant associations between the prevalence of MVCs and POAG severity when severity was categorized by the MD of the better eye or the IVF MD. Crabb et al. monitored patients' eye movements during driving simulations and reported that deterioration in the superior peripheral area of the binocular IVF could affect driving performance [24]. Glen et al. reported that the response rate for detecting hazards in a series of real-life driving films fell significantly when viewing films with a superior visual field defect, compared with an inferior visual field defect (*P* < 0.001) [25]. It has also been reported that specific VF regions are important for different tasks and affect hand-eye coordination [26], postural stability [27], risk of falling [13], and risk of fractures [28]. Further studies should be performed to investigate relationships between pointwise VF sensitivities and MVC history. Another possible explanation is that the number of participants classified as severe is simply too small to detect a significant result.

### 4.1. Strength of Our Study

To the best of our knowledge, we are the first group to report an association between glaucoma severity and MVCs in Japan and even in Asia. We have previously reported that severe glaucoma is associated with MVCs in Japan. However, the previous study was a single center study with 12 MVCs, relatively small number. In contrast, the total number of MVCs analyzed in our current study is 75, which allowed us to generate more robust results than in our previous study. Another important distinction from the previous study is that we clearly define MVCs in the questionnaire in this current study. Furthermore, we asked for history of MVCs in past 10 years in the previous study, while in the current study we asked for history of MVCs in only the past 5 years. Therefore, recall bias may be reduced in the current study. Finally, in our current study, we found novel factors to be associated with MVCs, including long distance driving and younger age, associations that were not detected in our previous study.

### 4.2. Study Limitations

The greatest limitation of our study is the use of self-reported MVCs, which may have a recall bias, as a main variable [29]. However, Marottoli et al. reported that compared with state-reported MVCs, self-reported MVCs provide sufficient information to assess crashes [30]. A reluctance to provide information may have affected the self-reported data; subjects with glaucoma who were followed up for a long time by the same doctor may have hesitated to provide a full history of MVCs and thus biased the result.

Another limitation is that we did not evaluate whether subjects were at fault in the MVCs they reported, which would be an important piece of information for this study. However, fault can be difficult to define in a car accident, especially in this type of self-reported questionnaire, and people are likely to answer that they were not responsible for MVCs. Therefore, in this study, we did not ask who was at fault in the MVCs.

The present study has some other limitations. Ophthalmic data were obtained after the MVCs had occurred, typically after an interval of up to 5 years, and the subjects' visual field defects may have worsened during that time period. This may have reduced the accuracy of our results. A causal association between MVCs and visual field defects was not confirmed in this cross-sectional study. A normal visual field was confirmed in control subjects by a fundoscopic evaluation of the optic disc, not by visual field testing. MVCs were not precisely defined in the questionnaire. These results may be partly dependent on the imbalance of group sizes when stratifying according to worse eye/best eye/IVF MD. Therefore, we cannot conclude that better eye MD and IVF MD are not associated with MVCs in this study. Increasing the sample sizes in severe glaucoma group categorized by better eye MD or IVF MD may give a different result.

Our study shows a significant association between the severity of glaucomatous visual field defects in the worse eye and MVCs. The association between MVCs and glaucoma severity is worthy of further investigation.

## Figures and Tables

**Table 1:** Demographic characteristics grouped by MD in the worse eye.

	Controls	Mild glaucoma	Moderate glaucoma	Severe glaucoma	*P* value
Number	187	92	60	47	
Age (years)	65.1 ± 10.8	63.5 ± 9.1	65.8 ± 9.1	63.7 ± 10.6	0.45^a^
Sex (M/F)	108/79	62/30	43/17	31/16	0.17^b^
Alcohol drinker	90 (48.1%)	49 (53.3%)	30 (50.0%)	25 (53.2%)	0.94^b^
Current smoker	24 (12.8%)	13 (14.1%)	10 (16.7%)	9 (19.1%)	0.69^b^
Diabetes mellitus	22 (11.8%)	19 (20.7%)	11 (18.3%)	4 (8.5%)	0.11^c^
Number of comorbid illnesses	0.74 ± 0.77	0.73 ± 0.81	0.77 ± 0.79	0.66 ± 0.82	0.82^d^
Driving history (years)	37.8 ± 11.2	37.6 ± 10.8	39.4 ± 9.0	36.9 ± 11.1	0.64^a^
Distance (km) driven weekly	77 ± 113	88 ± 127	133 ± 223	76 ± 102	0.06^d^
BCVA, better eye (LogMAR)	0.00 ± 0.02	0.00 ± 0.01	0.01 ± 0.02	0.01 ± 0.03	0.26^d^
BCVA, worse eye (LogMAR)	0.02 ± 0.04	0.01 ± 0.02	0.02 ± 0.05	0.03 ± 0.05	0.04^d^
MD, better eye (dB)	NA	−1.0 ± 1.5	−3.2 ± 2.7	−7.2 ± 5.9	<0.001^a^
MD, worse eye (dB)	NA	−2.8 ± 1.6	−9.0 ± 1.6	−18.1 ± 5.0	<0.001^a^

M: male, F: female, BCVA: best corrected visual acuity, LogMAR: logarithm of the minimum angle of resolution, MD: mean deviation, and dB: decibels.

^a^ANOVA, ^b^chi-square test, ^c^Fisher's exact test, and ^d^Kruskal-Wallis test.

**Table 2:** Demographic characteristics, grouped by MD in the better eye.

	Control	Mild glaucoma	Moderate glaucoma	Severe glaucoma	*P* value
Number	187	163	24	12	
Age (years)	65.1 ± 10.8	64.4 ± 9.1	64.2 ± 11.2	63.3 ± 11.9	0.87^a^
Sex (M/F)	108/79	110/53	17/7	9/3	0.19^b^
Alcohol drinker	90 (48.1%)	79 (48.4%)	14 (58.3%)	11 (91.7%)	0.02^b^
Current smoker	24 (12.8%)	23 (14.1%)	5 (20.8%)	4 (33.3%)	0.18^b^
Diabetes mellitus	22 (11.8%)	29 (20.7%)	4 (18.3%)	1 (8.5%)	0.11^b^
Number of comorbid illnesses	0.74 ± 0.77	0.71 ± 0.76	0.96 ± 1.06	0.50 ± 0.80	0.51^c^
History of driving (years)	37.8 ± 11.2	38.4 ± 10.3	35.4 ± 9.5	37.1 ± 12.3	0.63^a^
Distance (km) driven weekly	77 ± 113	102 ± 173	71 ± 85	104 ± 143	0.34^a^
BCVA, better eye (LogMAR)	0.00 ± 0.02	0.00 ± 0.01	0.01 ± 0.03	0.01 ± 0.03	0.84^c^
BCVA, worse eye (LogMAR)	0.02 ± 0.04	0.01 ± 0.04	0.02 ± 0.05	0.03 ± 0.06	0.62^c^
MD, better eye (dB)	NA	−1.5 ± 1.8	−8.0 ± 1.6	−15.4 ± 2.4	<0.001^a^
MD, worse eye (dB)	NA	−6.7 ± 5.8	−13.2 ± 5.1	−19.9 ± 4.1	<0.001^a^

M: male, F: female, BCVA: best corrected visual acuity, LogMAR: logarithm of the minimum angle of resolution, MD: mean deviation, dB: decibels, and NA: not applicable.

^a^ANOVA; ^b^Fisher's exact test; ^c^Kruskal-Wallis test.

**Table 3:** Demographic characteristics, grouped by IVF MD.

	Control	Mild glaucoma	Moderate glaucoma	Severe glaucoma	*P* value
Number^a^	187	165	20	6	
Age (years)	65.1 ± 10.8	64.0 ± 9.2	67.8 ± 10.1	54.5 ± 12.4	0.03^b^
Sex (M/F)	108/79	113/52	13/7	4/2	0.20^c^
Alcohol drinker	93 (49.7%)	82 (49.7%)	15 (75.0%)	5 (83.3%)	0.06^c^
Current smoker	24 (12.8%)	23 (13.9%)	7 (35.0%)	2 (33.3%)	0.03^c^
Diabetes mellitus	22 (11.8%)	29 (20.7%)	2 (10.0%)	0 (0.0%)	0.37^c^
Number of comorbid illnesses	0.74 ± 0.77	0.72 ± 0.79	0.75 ± 0.97	0.33 ± 0.82	0.50^d^
History of driving (years)	37.8 ± 11.2	38.1 ± 10.5	37.5 ± 9.7	31.0 ± 12.2	0.47^b^
Distance (km) driven weekly	77 ± 113	98 ± 150	74 ± 103	101 ± 150	0.87^d^
BCVA, better eye (LogMAR)	0.00 ± 0.02	0.00 ± 0.01	0.02 ± 0.04	0.00 ± 0.00	0.02^d^
BCVA, worse eye (LogMAR)	0.02 ± 0.04	0.01 ± 0.04	0.04 ± 0.06	0.03 ± 0.06	0.06^d^
MD, better eye (dB)	NA	−1.8 ± 2.2	−9.3 ± 4.3	−14.7 ± 2.6	<0.001^b^
MD, worse eye (dB)	NA	−6.9 ± 5.7	−15.2 ± 5.5	−19.3 ± 5.2	<0.001^b^

M: male, F: female, BCVA: best corrected visual acuity, LogMAR: logarithm of the minimum angle of resolution, MD: mean deviation, dB: decibels, and NA: not applicable.

^a^Table shows results for 378 subjects (we were unable to obtain IVF data for 8 POAG subjects); ^b^ANOVA; ^c^Fisher's exact test; ^d^Kruskal-Wallis test.

**Table 4:** Prevalence of MVCs in subjects with POAG, grouped by MD in the better eye, worse eye, or IVF.

		Control	Mild glaucoma	Moderate glaucoma	Severe glaucoma	*P* value
	MD, worse eye	30/187	17/92	14/60	14/47	0.025^a^
		(16.0%)	(18.5%)	(23.3%)	(29.8%)	
Severity, grouped by	MD, better eye	30/187	36/163	6/24	3/12	0.12^a^
		(16.0%)	(22.1%)	(25.0%)	(25.0%)	
	MD, IVF	30/187	41/165	3/20	1/6	0.27^a^
		(16.0%)	(24.8%)	(15.0%)	(16.7%)	

MVC: motor vehicle collision, POAG: primary open-angle glaucoma, MD: mean deviation, and IVF: integrated visual field.

^a^Cochran-Armitage trend test.

**Table 5:** Glaucoma severity and MVC prevalence.

Severity grouped by		Mild glaucoma	Moderate glaucoma	Severe glaucoma
MD, worse eye	OR^a^	1.07	1.44	2.28
	[95% CI]	[0.55–2.10]	[0.68–3.08]	[1.07–4.88]
	*P* value	0.84	0.34	0.03^∗^

MD, better eye	OR^a^	1.36	1.82	1.65
	[95% CI]	[0.78–2.37]	[0.65–5.11]	[0.39–6.87]
	*P* value	0.28	0.26	0.49

IVF MD^†^	OR^a^	1.59	1.01	NA
	[95% CI]	[0.92–2.74]	[0.31–3.28]	
	*P* value	0.10	0.99	

^a^Multivariable logistic regression analysis adjusted for age, sex, the presence of diabetes mellitus, the proportion of alcohol drinkers, worse eye BCVA, and distance driven per week (controls used as reference).

MVC: motor vehicle collision, BCVA: best corrected visual acuity, MD: mean deviation, IVF: integrated visual field, OR: odds ratio, and CI: confidence interval.

^†^Only 6 subjects with severe glaucoma were categorized by IVF MD; these 6 were analysed with the moderate glaucoma group (logistic regression results did not differ when these two groups were analysed separately).

^∗^ *P* < 0.05.
